# Identifying the research gap of zoonotic disease in displacement: a systematic review

**DOI:** 10.1186/s41256-021-00205-3

**Published:** 2021-07-16

**Authors:** Dorien Hanneke Braam, Freya Louise Jephcott, James Lionel Norman Wood

**Affiliations:** Disease Dynamics Unit, Department of Veterinary Medicine, University of Cambridge, Cambridge, UK

**Keywords:** Zoonotic diseases, Zoonoses, Forced migration, Displacement, One health, Humanitarian emergencies, Syndemics

## Abstract

**Background:**

Outbreaks of zoonotic diseases that transmit between animals and humans, against a backdrop of increasing levels of forced migration, present a major challenge to global public health. This review provides an overview of the currently available evidence of how displacement may affect zoonotic disease and pathogen transmission, with the aim to better understand how to protect health and resilience of displaced and host populations.

**Methods:**

A systematic review was conducted aligned with the Preferred Reporting Items for Systematic Reviews and Meta-Analyses (PRISMA) reporting guidelines. Between December 2019 - February 2020, PubMed, Web of Science, PLoS, ProQuest, Science Direct and JSTOR were searched for literature. Studies were included based on a focus on zoonotic disease risks in displacement and/or humanitarian emergencies, and relevance in terms of livestock dependency of the displaced populations. Evidence was synthesised in form of a table and thematic analysis.

**Results:**

Of all records, 78 papers were selected for inclusion. Among the included studies, the majority were based on secondary data, including literature reviews (n=43) and case studies (n=5), while the majority of papers covered wide geographical areas such as the Global South (n=17) and Africa (n=20). The review shows significant gaps in the literature, which is specifically lacking primary data on zoonotic diseases in displacement. Risk factors for the transmission of zoonoses in displacement are based on generic infectious disease risks, which include the loss of health services, increased population density, changes in environment, reduced quality of living conditions and socio-economic factors. Regardless of the presence of these disease drivers during forced migration however, there is little evidence of large-scale zoonotic disease outbreaks linked directly to livestock in displacement.

**Conclusion:**

Due to the lack of primary research, the complex interlinkages of factors affecting zoonotic pathogen transmission in displacement remain unclear. While the presence of animals may increase the burden of zoonotic pathogens, maintaining access to livestock may improve livelihoods, nutrition and mental health, with the potential to reduce people’s vulnerability to disease. Further primary interdisciplinary and multi-sectoral research is urgently required to address the evidence gaps identified in this review to support policy and program development.

**Supplementary Information:**

The online version contains supplementary material available at 10.1186/s41256-021-00205-3.

## Background

Research shows that most emerging infectious diseases in humans have animal origins, either originating in domestic animals or wildlife [1], while neglected and endemic zoonoses, continuously transmitted between livestock and humans, are a significant burden to public health and livelihoods [2]. The transmission of zoonotic pathogens depends on complex interactions between susceptibility, periodicity and anthropogenic activities [3], influenced by a range of ecological, political and socio-economic drivers [3–5]. Poverty and low socio-economic status are among the most important determinants of people’s vulnerability to disease [6], with people whose livelihoods are affected by conflict or disasters therefore considered to be at an even higher risk. Humanitarian emergencies may result in the displacement of human and livestock populations. Movement is associated with increased mixing of displaced and host populations’ and their livestock, and increased contact between domestic animals, wildlife and humans, which risks increased disease transmission between species. Where animals and humans move into new environments, they may face new pathogens and vectors prevalent among local animal and human populations – the ‘host’ population to the displaced, against which they lack immunity. Health services and staff may be affected or become displaced themselves, hampering an organized response, exacerbating zoonotic disease outbreaks [6].

The number of displaced people is consistently growing [7], increasingly caused by environmental drivers [8]. Many of these forced migrants move in regions dependent on agriculture and livestock [9]. As livestock are relatively mobile, these are often among the few assets people bring along, however currently animals are largely banned in formal relief camps, due to the hypothetical increased risk of zoonotic disease. In response, displaced people may abandon or sell their animals before or during displacement, affecting people’s nutrition, psychosocial health, and ability to rebuild livelihoods [10]. The lack of access to formal relief camps of livestock because of zoonotic disease concerns acts as a deterrent from accessing services, as households or individual family members may opt to stay behind with the herds [11]. Lacking the provision of protection, water and feed for their animals in formal humanitarian responses, owners may adapt by letting their herds graze among host communities’ livestock, or encroach on wildlife habitat, increasing the risk of introduction of zoonotic pathogens to naïve populations, further increasing the risk of zoonotic disease [12].

Due to a lack of primary research addressing zoonoses in displacement contexts, zoonotic disease dynamics and related risks in displacement are not well understood. The purpose of this literature review is to identify research gaps and analyse the current available evidence on zoonotic disease in displaced populations.

## Methods

### Search strategy

This literature review was conducted based on the Preferred Reporting Items for Systematic Reviews and meta-Analysis (PRISMA) statement [13]. The database search was carried out between December 2019–February 2020, using the databases of PubMed, Web of Science, PLoS, ProQuest, Science Direct and JSTOR. To capture all available publications discussing zoonoses in displacement, the search strategy used a variety and combinations of search terms related to displacement, zoonotic diseases and humanitarian emergencies. No parameters were set regarding time period.

### Study selection

Papers were only considered if the full text was available in English, thereby introducing a potential publication bias. All available abstracts were screened and included based on their focus on zoonotic disease risks in displacement and/or humanitarian emergencies, and relevance in terms of livestock dependency of the displaced populations. Duplicates were excluded from the review. The most important (grey) literature references within the literature were included in screening, based on the number of times these were referenced in various literature sources.

### Quality assessment

The quality of eligible studies was assessed through a full-text review, evaluating the quality of literature reviews and primary data using the Critical Appraisal Skills Programme (CASP) model. Any disagreements were resolved through discussion.

### Data extraction and analysis

**Fig. 1:**
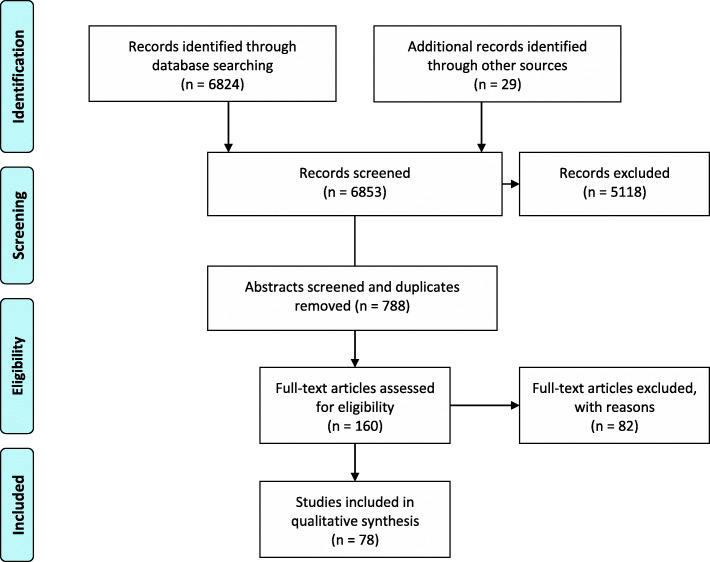
PRISMA systematic review protocol diagram for the literature selection and narrative synthesis

All included papers were subject to a full-text analysis using a thematical analysis to develop an evidence matrix, which captured relevant data from each source using the main themes emerging from the literature. Themes captured included references to animal movement, causes and type of displacement, the effect of displacement on socio-economic, environmental, and biological factors. All literature was screened with a focus on the impact of (livestock) displacement on health systems, infectious disease outbreaks, disease dynamics and references to zoonoses in particular. Eventually, 78 papers were included in the systematic literature review for qualitative analysis (Fig. 1).

In this literature review we provide an overview of the currently available evidence of 1) zoonotic diseases associated with displacement contexts, and 2) drivers during displacement affecting zoonotic pathogen transmission risks, followed by a discussion addressing 3) gaps in the literature, and 4) current risk mitigation measures, concluding with entry points for further research to increase understanding on how to protect health, livelihoods and resilience of displaced populations, host communities and livestock.

## Results

**Fig. 2:**
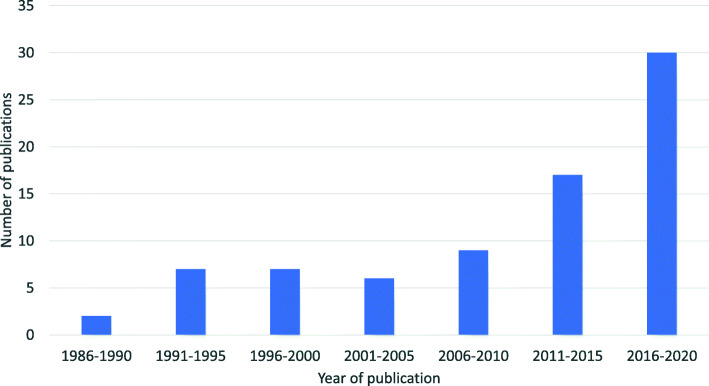
Volume of relevant publications since 1986

The volume of publications identified in the review increases over time, with most of the included literature published within the last five years (Fig. 2).

**Fig. 3:**
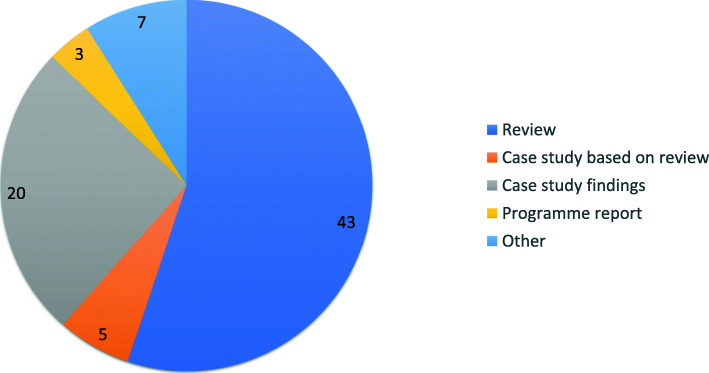
Type of publications included in the literature review

Our review shows that there is a lack of primary research data. Over 55% of publications are literature reviews (n=43) or case studies based on secondary data (n=5) often with a qualitative focus. Case study findings through primary research were discussed in 20 papers, while 3 were program outcome reports. The other documents included dynamic disease models and United Nations (UN) documents (Fig. 3).

No publication focused on the specific risk of zoonoses related to livestock movement during displacement.

**Fig. 4:**
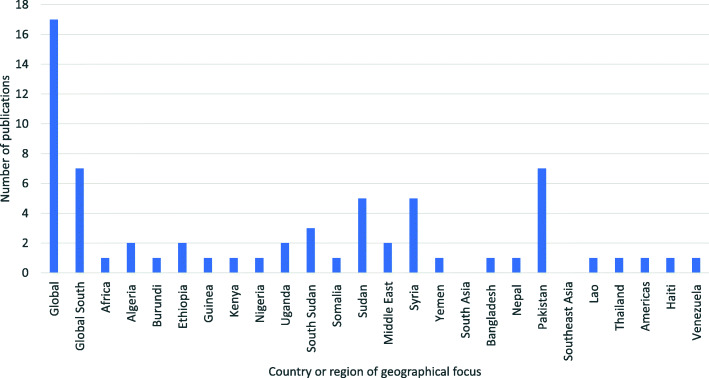
The number of publications focusing on specific geographical regions and countries

Geographically, studies included in the review are primarily global reviews (n=17), or focus on the ‘Global South’, a region disproportionally affected by forced displacement. In addition to regional reviews (n=7), papers cover individual countries in Africa (n=20), South Asia (n=9) and the Middle East (n=8), all areas with high levels of displacement and livestock dependency, with a growing body of literature discussing the adverse impact of the conflict in Syria (n=5) []. Papers focusing on Pakistan primarily discuss Afghan refugee health, which remains one of the largest refugee populations in the world. Three papers focus on South America and two on Southeast Asia, but no relevant literature covered East Asia or the Pacific (Fig. 4).

Most publications focus on general infectious disease risks in humanitarian emergencies, which sometimes include zoonotic diseases or symptoms, which may be attributed to zoonoses. There is a gap in the literature related to livestock in displacement and the associated risk of zoonotic diseases, resulting in assumptions regarding risk factors and transmission routes.

### Diseases associated with humanitarian emergencies

**Fig. 5:**
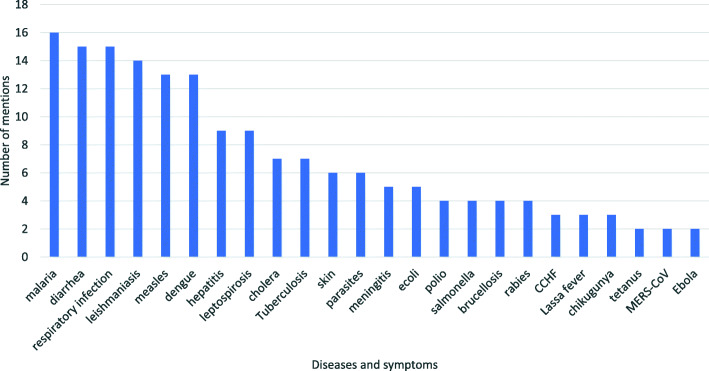
Symptoms and diseases associated with humanitarian emergencies (as referred to in > 2 independent reviewed articles)

While disasters and conflict do not directly cause infectious diseases [14, 15], a disaster or conflict can ‘eliminate pre-existing barriers separating hosts and agents’ through the destruction of physical structures [16], introducing pathogens to naive populations. Injuries can lead to infections where pathogens are present [16, 17]. Watson et al [18] note that vector-borne, water and crowding-related diseases are the most common causes of epidemics after disasters, with up to 75 percent of mortality due to both zoonotic and non-zoonotic diseases. Regular occurring infectious diseases and symptoms following emergencies are diarrhea, malaria, measles, pneumonia, upper and lower respiratory tract infections, skin diseases, tetanus and anaemia, several of which may be attributed to zoonoses [14, 19, 20]. Diarrhea is one of the main causes of morbidity and mortality in emergencies, especially among young children [15, 21]. In flood-related disasters eye infections, leptospirosis, hepatitis and leishmaniasis are also common (Fig. 5) [22, 23].

Among the variety of symptoms and infectious diseases identified in the literature are a number of zoonotic diseases and/or symptoms which may be caused by zoonoses, although there remains a lack of primary data. Heath et al [24] identified diseases potentially affecting livestock following disasters including parasites, respiratory infections and skin diseases, some of which zoonotic. Human disease outbreaks associated with population displacement include Ebola, Lassa fever and tuberculosis [17, 25, 26] (Table 1).

**Table 1:** Zoonotic diseases of concern in humanitarian emergencies and displacement (identified in the literature reviewed), disease data from: WHO communicable disease control in emergencies, A field manual [28]

Zoonoses	Symptoms in humans	Transmission	Main risk factors	Geographical locations
Contamination and direct transmission				
Salmonellosis (caused by non-typhoidal Salmonella)	acute abdominal pain, diarrhoea, nausea, fever, and sometimes vomiting	contaminated water and food, livestock products, contact with infected animals	living conditions: lack of hygiene, especially of food	Asia [16], Bangladesh [27], Indonesia [17, 18], Syria [28], EU [5, 29], Italy [29], US [18, 30], Haiti [15].
Foodborne infections (caused by *E. coli*), respiratory illness, pneumonia	diarrhea, vomiting, fever, respiratory disease symptoms	contaminated water and food, livestock products, contact with infected animals	living conditions: lack of hygiene, especially of food	Bangladesh [18, 27, 31, 32], Thailand [16], Syria [28], EU [29], Denmark [29], US [30], Haiti [15].
Hepatitis E	yellow skin, secretions, tiredness, fever, vomiting, nausea, abdominal pain, loss of appetite; liver failure, death during pregnancy	human sewage contaminated food and water; eating undercooked pig meat	living conditions: lack of hygiene	Sub-Saharan Africa [16], Ethiopia [14], Kenya [14], Somalia [14], Sudan [27, 32–36], South Sudan [34], Asia [16], Bangladesh [31], India [32], Nepal [37], Pakistan [17, 18, 22, 37], Indonesia [17, 18], Thailand [38], Turkey [29]
Leptospirosis	fever, headache, nausea, loss of appetite, jaundice, swollen limbs, chest pain, shortness of breath, coughing blood	contaminated soil and water, animal urine	injuries, skin lesions; occupational exposure	Southeast Asia [38], Philippines [27], Thailand [17, 38], China [17], India [17, 18, 32], Nepal [37], Pakistan [22], Taiwan [17, 18], Puerto Rico [16], Central and South America [39] Argentina [18], Brazil [18], Nicaragua [39], Caribbean [24], Haiti [15, 39], Austria [29], Bulgaria [29], Germany [29], France [29], Russia [29].
Bovine tuberculosis	weakness, loss of appetite, weight loss, fluctuating fever, intermittent cough, diarrhoea, large prominent lymph nodes	eating or drinking contaminated, unpasteurized dairy products direct contact with infected animals or contaminated tissues during slaughter	animal husbandry; living conditions; occupational exposure; wildlife reservoirs	Africa [40], Nigeria [12].
Brucellosis	fluctuating fever, body ache, weight loss, weakness, abdonimal pain	contact with aborted foetuses, vaginal fluids, placentae, placental fluids, urine, semen, feces and hygroma fluids fomites, milk	occupational exposure; ingesting unpasteurised dairy products	Africa [40], Algeria [41], Nigeria [12], Tanzania [2], Somalia [42], Mongolia [2], Nepal [37], Syria [28].
Rabies	rapidly progessive fatal encephalitis, preceded by generalised arousal or hyperexcitability and hydrophobia; progressive lower motor neuron weakness, paralysis	animal bites (especially dogs)	free roaming dogs; wildlife, rarely pets	Africa [3, 40], Algeria [41], Chad [6], Malawi [2], Niger [6], Indonesia [24], Myanmar [6], Nepal [37], Thailand [38], Syria [28], Cuba [24], Haiti [15, 39].
Middle East Respiratory Syndrome (MERS	fever, cough, shortness of breath, pneumonia, gastrointestinal symptoms, diarrhoea; respiratory failure	transmission in unprotected healthcare settings; dromedary contact, undercooked meat	lack of quality healthcare	Eastern Meditteranean Region [43, 44], Middle East [45].
Lassa Hemorrhagic Fever	fever, malaise, headache, sore throat, muscle pain, chest pain, nausea, vomiting, diarrhea, cough, abdominal pain; bleeding, low blood pressure and death	contaminated food or other items by infected rats; transmission in unprotected healthcare settings	living conditions, lack of hygiene; lack of quality healthcare	Africa [25], West Africa [5, 36, 46], Guinea [3], Liberia, Sierra Leone [3, 36].
Ebola Virus Disease	fever, fatigue, muscle pain, headache, sore throat, vomiting, diarrhoea, rash, impaired kidney and liver function; internal and external bleeding, low white blood cell and platelet counts, elevated liver enzymes	human to human bodily fluids contact; close contact with the blood, secretions, organs or other bodily fluids of infected animals	living conditions and poor hygiene, lack of quality healthcare, pregnancy, lactation, burial practices	Africa [6, 7, 25], West Africa [5, 29, 43, 45, 47, 48], Gabon [30], Guinea [43], Ivory Coast [25], Liberia [25, 43], Sierra Leone [43], DRC [25, 36, 49], Sudan [25].
Parasites				
Endo-parasites, including *Opistorchis*, *Echinostoma*, *Strongyloides*, *Taenia*, *Sarcocystis*, hookworm [42], *Giardia*, *Ascaris*, *Trichuris*, *Echinococcus* [44].	failure to thrive, skin bumps, rashes, weight loss, increased appetite, abdominal pain, diarrhea, and vomiting, sleeping problems, anaemia, aches and pains, allergies, weakness, malaise, fever	contaminated food and water, bites, contaminated soil	high population density, poor sanitation	Angola, Burkina Faso, Central African Republic, DRC, Equaltorial Guinea, Gabon, Liberia, South Sudan [6], Eastern Africa [40], Ethiopia [50], Eritrea, Swaziland, Zambia, Zimbabwe, Afghanistan, China, Laos, Malaysia, Mongolia, Tajikistan, Papua New Guinea, Thailand [6, 38], Kiribati, Marschall Islands, Guatemala, Peru, Jamaica [6].
Vector-borne				
Leishmaniasis	cutaneous leishmaniasis: skin lesions, nodules, or papules; visceral leishmaniasis: fever, weight loss, enlargement of the spleen and liver - untreated typically fatal	Leishmania parasite through bite of female phlebotomine sand fly which feed on blood; 70 animal species are natural reservoir hosts, including humans	lack of vector control; environmental changes, and urbanization; malnutrition, population displacement, poor housing/ sanitation, health status, poverty	Ethiopia [50], South Sudan [51, 52], Sudan [53–55], Northern Africa Middle East, Iraq, Jordan, Lebanon, Libya, Saudi Arabia [56], Syria [28, 43, 56, 57], Tunisia, Turkey, Yemen [6], Afghanistan [54, 56], Pakistan [22, 56, 58], Nepal [37], Brazil [52], Colombia, Venezuela [54, 59].
Crimean-Congo haemorrhagic fever (CCHF)	fever, myalgia, dizziness, neck pain, stiffness, backache, headache, sore eyes and photophobia, nausea, vomiting, diarrhoea, abdominal pain, sore throat; mood swings, confusion; sleepiness, depression lassitude, liver enlargement, tachycardia, lymphadenopathy, petechial rash; hepatitis, liver failure	tick bites, contact with infected livestock	occupational exposure; population movement	Africa, Asia, Europe, Middle East [60], Eastern Mediterranean Region [43], Turkey [28, 60], Afghanistan, Iran [44], Pakistan [61, 62].
Rift Valley Fever (RVF)	range from mild flu-like illness to severe lethal haemorrhagic fever	contact with blood or organs of infected livestock; mosquito bites; unpasteurized milk	occupational exposure; lack of vector control	Africa [40, 60, 63], Egypt [63], Kenya [24, 45, 63], Somalia [59, 63], Sudan [63], Tanzania [59], Eastern Mediterranean Region [43], Asia [60].

### Disease drivers in displacement

Displacement as a result of disasters and conflict is considered a major risk factor for pathogen transmission, including zoonoses [6, 18, 48, 51, 54, 57]. Mortality among refugees is reported to be as much as 60 times a population’s pre-disaster baseline [15]. Writing about the risks of displacement to Lassa fever outbreaks, Lalis et al [46] acknowledge however that other socio-economic and political factors may influence health outcomes. Rather than considering displacement as an independent risk factor, human and animal movement are more likely to exacerbate a range of other disease drivers.

#### Health systems

The breakdown of health systems and related infrastructure is considered a major risk factor for pathogen transmission during emergencies and displacement [6, 14, 19, 27, 36, 64], affecting a population’s health status and immunization coverage, increasing susceptibility to disease [49]. Healthcare and veterinary services may deteriorate or get overwhelmed [14], and public expenditure into the system often decreases [25, 35, 37]. Medical staff become exhausted, injured or displaced themselves, while a loss of management hampers the distribution of resources, supplies and equipment [34]. An interruption in health services affects surveillance, prevention, diagnosis and treatment and control programmes including vaccinations, quarantine and vector control [15, 44, 53, 55], the provision of medication and follow-up [19, 64]. Clinics and other facilities, such as laboratories, may be destroyed or otherwise become inaccessible [18, 44], while cold chains for vaccine and medicine storage and transfer become interrupted or unavailable [34, 51].

Decreased immunization among displaced populations, or immunization gaps between refugees and the host population, increases the risk of vaccine preventable diseases [34, 64], although most of these are not zoonotic. A lack of quarantine and immunization of new arrivals may cause disease outbreaks among displaced and host populations [65]. The collapse of veterinary public health systems in Syria was associated with an increase in zoonotic leishmaniasis, brucellosis and rabies cases [28], including in neighbouring countries, as shifting control of geographical locations between government and opposing forces in Syria challenged disease surveillance and control [64]. During Venezuela’s recent displacement crisis vector-borne diseases re-emerged due to the lack of control programmes, resulting in outbreaks in neighbouring countries [59]. Meanwhile, the lack of vaccinations and surveillance led to outbreaks of infectious diseases among displaced and returned populations in Pakistan after the floods in 2010, including the zoonoses Crimean-Congo haemorrhagic fever [62].

#### Environment

Pathogen prevalence, available vectors and suitable hosts determine the risk of infectious disease outbreaks [27, 61]. Humanitarian emergencies may alter the natural environment, thereby affecting pathogen and vector ecology, including selection pressure, development, survival, modification and transmission rates [30, 38, 63]. Structural damage during conflict and disasters has shown an increase in rodent populations and associated diseases [36]. Displacement may modify the environment through deforestation, the construction of settlements and irrigation, all affecting pathogen and vector dynamics [19, 38]. Lassa fever outbreaks for instance occurred among populations of refugee camps in West Africa due to ecological changes, impacting the size and genetic variability of the rodent and pathogen populations attributed to forest and habitat destruction, in combination with poor living and food storage conditions attracting rodents [36, 46].

Population displacement changes the geographic distribution of susceptible populations [26] and pathogens [38], altering the rates and nature of contact between human and animal populations, increasing the risks of bites and zoonotic diseases [1, 27, 39]. Livestock movement further extends the range of pathogens and vectors threatening naïve host populations [38, 42, 66].

Displaced populations may enter new ecological zones without immunity to local pathogens [10, 19, 38, 67], or introduce pathogens to naive host populations by mixing infected and susceptible herds with different levels of pre-existing immunity and immune responses [6, 40, 52, 59]. Afghan refugee movements for instance are linked to the reintroduction of cutaneous leishmaniasis to Pakistan into areas where the sandfly vector is endemic [58, 68], as well as other zoonoses [61]. Similarly, the disease resurfaced in neighbouring countries to Syria following the outbreak of conflict, associated with population movements into previously uninhabited sandfly habitats [28, 56, 57].

#### Population density

Overcrowded camps and inadequate facilities are major risks to health, including interspecies and intraspecies infection [18, 19, 25, 27, 31, 34, 36, 39, 42, 50, 62]. As the transmission of zoonotic pathogens is linked to the close association of humans and their livestock [5, 40, 60], these risks increase in areas where animals and humans share compounds in densely populated areas [10, 36, 54, 61]. Sedentary conditions in relief camps and informal settlements further increases the risk of intraspecies zoonotic pathogen transmission, once the disease has become endemic among the human population [32, 38, 42], as population size and density affects the probability of pathogens to infect susceptible hosts [17, 47, 58, 60, 65].

#### Water and sanitation

Standing water amid destroyed housing and infrastructure can create new breeding sites for vectors [16, 43, 67], while flooding may cause sewage overflow, contaminating the water supply [29], causing favorable conditions, for instance for leptospirosis transmission [29, 39]. Animal and human feces may contaminate water and food sources, causing disease [16, 20, 22, 25, 31, 40], such as gastrointestinal infections and Hepatitis A and E [16]. Due to the increased sharing of water sources among domestic animals and humans zoonotic parasitic infections risk is greater during displacement [40]. In Darfur, the lack of a clean water source was an important factor in an outbreak of Hepatitis E among displaced people [33]. Shears and Lusty [19] note however that the impact of improved water supply and sanitation during displacement is minimal if overcrowding is not addressed, as pollution may still occur further down the distribution chain.

#### Living conditions

Services in relief camps are often limited due to funding, logistical and sourcing constraints [31]. Inadequate shelter may increase the risk of transmission of zoonotic pathogens, as certain shelter types may not be suitable for vector control, for instance wooden huts cannot be treated with insecticide [19]. Brooker et al [58] showed that shelter materials impacted the risk of cutaneous leishmaniasis. Meanwhile, inadequate living conditions affecting human-animal interactions may pose risks to pathogen transmission pathways beyond zoonoses, as the lack of distance between animal and human hosts may cause an increase in prevalence of diseases such as malaria [63].

Broglia et al [69] identify the lack of hygiene as most problematic feature of animal husbandry in refugee camps, caused by inappropriate shelters and a change in husbandry practices. Animals may act as an additional feeding source for sandfly and other vectors [58], while the presence of dogs increases the risk of rabies [54]. Vector borne diseases in north west Pakistan have been ascribed to refugees bringing their livestock from Afghanistan into poor and dense living conditions [61], while keeping ruminants inside the compound at night for security increased people's risk of being bitten by Anopheles mosquitoes and malaria [70].

#### Nutrition

In disasters and complex emergencies, livelihoods may be lost and regular food supply disrupted due to a decline in agricultural input and output, diversion and loss [14, 25]. Malnutrition of both animals and humans is common, and an important risk factor increasing susceptibility to, and the severity of,zoonotic disease [19, 31, 34, 38, 51, 65].

Usually situated near roads and water sources, displacement camps and informal settlements are often established in marginalized areas lacking vegetation and agriculture, which may result in malnutrition and metabolic disorders in livestock, exacerbated by no-grazing policies in camps [69]. Compromised immunity of both animals and humans through exhaustion from displacement, untreated parasites and gastrointestinal infections further affect malnutrition [31, 50].

#### Socio-economic

As socio-economic inequities and poverty are associated with poor health [6, 39, 71], disasters and displacement affect the availability of education, labour and livelihoods, further exacerbating poverty [6]. Displaced populations often face structural discrimination and violence, including a lack of equitable access to services [72]. Furthermore, displaced communities often live in marginalized geographical locations, with limited resources [73]. In areas where refugees move into poor host communities, disease outbreaks are more likely, for instance communities along the Afghan-Pakistan border bear the brunt of vector-borne diseases caused by displacement [61].

## Discussion

The literature review confirms Hammer et al [47] who noted that issues described in the literature around infectious diseases in complex emergencies have been 'poorly evidenced, not contextualised and not considered with respect to interaction effects'. While our review shows an increase in relevant literature in the past five years, which may be associated by a global increase in displaced populations, as well as renewed interest in, and emerging interdisciplinary approaches to zoonotic diseases, there remains a lack of primary, field-based evidence on zoonotic disease risks during displacement. Researchers point out the need for more research on zoonoses [1], interactions between population movement and infectious diseases [47, 54], interspecies interactions between humans and animals, including during displacement [41, 74, 75], and social and epidemiological factors [45]. There is currently no data available on these complex interlinkages however, and any positive effects displaced animals may have on the epidemiology and dynamics of zoonoses [10].

Disease outbreaks depend on the presence of contagious pathogens and susceptible hosts [27], and transmission is influenced by the health and immunity status of the displaced and host human and animal populations and their mixing [18]. Most risk factors do not result in disease outbreaks in isolation. While poverty and malnutrition are associated with general ill health, the availability of quarantine and vaccinations determine the effectiveness of infectious disease control. Even where services are available, tradition and social pressure determines whether people access resources [10]. The collapse of health services and infrastructure is a major determinant for infectious disease risks in humanitarian emergencies, including zoonoses. Subsequent displacement affects vulnerability of displaced and host populations to vectors and pathogens by changing environmental conditions, increasing population density and reducing the quality of living conditions affecting hygiene [22]. Displaced populations are even more vulnerable to infectious disease due to malnutrition and long-term stress [36].

### Risk mitigation

Disease prevention and preparedness, surveillance, early monitoring of risk factors and epidemiology are especially relevant in displacement [19, 32, 35, 58, 61, 65]. To address infectious disease risks and compound hazards, the World Health Organization (WHO, 2005) recommends conducting assessments [17, 18, 47, 56], followed by prevention measures, improving water supply and sanitation, preventing overcrowding, promoting hygiene [14, 19, 22, 36, 37, 47], disease diagnoses, treatment and control, vaccination and immunization [14, 19, 34, 47, 62, 65]. The impact of these measures is not well studied however. Data on disease incidence, epidemiology and medical geography, ecology, distribution needs to be collected [36, 67], and include details of human behavior [5, 42, 45, 48, 76], in support of planning of camps [42, 49, 50].

While there is a lack of published evidence for the use of Livestock Emergency Guidelines and Standards (LEGS) or other standardized guidelines, some targeted livestock support programs are implemented in humanitarian emergencies, including vaccination campaigns and the provision of animal shelter [41, 76, 77]. Community based preparedness in camps and informal settlements improves animal husbandry and shelter [69], including the use of mosquito nets [31, 70]. Feeding programs are recommended to mitigate malnutrition, improving animal health [14, 21, 24]. Watson and Catley [78] provide examples of an integrated livestock emergency response system, combining feed, water and health interventions, or destocking with the provision of feed.

Without proper coordination and oversight however, zoonotic disease control may have unintended consequences. In Guinea the killing, collection and burying of rats was promoted in refugee camps to prevent Lassa outbreaks; however, this may not have completely stopped the consumption of rodents, as anecdotal evidence suggests that some residents considered this as ‘wasted food’ [46]. The lack of coordination between veterinary and public health actors affects public health [36], and is therefore one of the main requirements of LEGS and WHO’s field manual ‘communicable disease control in emergencies’ [21]. To mitigate the lack of veterinary services in disaster preparedness and responses, animal health specialists should get involved in the development of legislation and response plans [24].

To address the risk of zoonotic pathogen transmission during displacement, stakeholders need to address disease control, as well as political and socio-economic factors such as poverty and access to services. Public health and policy support needs to be interdisciplinary and multi-sectoral, and consider not only veterinary and public health, but also political, social and economic realities of displacement contexts, to enable durable solutions [3, 15, 30, 35, 39, 51, 62, 63, 73, 78].

## Conclusion

The knowledge gap of zoonoses in displacement may be ascribed to a lack of research into the epidemiology of specific diseases, as zoonoses are often difficult to diagnose, or may indicate that the presence of livestock has not proven to be as much of a risk factor as assumed. Instead, maintaining access to livestock may improve livelihoods, nutrition and mental health, with the potential to reduce people’s vulnerability to disease, providing a strong argument for allowing animals into relief camps if inadequate living conditions and sanitation are addressed appropriately. There is a projected increase in displacement due to environmental causes, particularly affecting areas dependent on agriculture and livestock. The role of livestock in displacement, its impact on host communities, and the potential benefits of maintaining displaced communities’ access to animals, in terms of livelihoods and health, need to be actively researched to better inform policies and programs related to health, livelihoods and human movement.

## Supplementary Information

**Additional file 1.**

## Data Availability

All data generated or analysed during this study are included in this published article.

## Abbreviations

LEGS: Livestock Emergency Guidelines and Standards
PRISMA: Preferred Reporting Items for Systematic Reviews and meta-Analysis
UN: United Nations
WHO: World Health Organization
