# Supplementary material for: Identifying the research gap of zoonotic disease in displacement: a systematic review
Source: Glob Health Res Policy. 2021 Jul 16;6:25. doi: 10.1186/s41256-021-00205-3 (PMC8283393; doi:10.1186/s41256-021-00205-3)
Supplement: Supplementary file 1 — Additional file 1. [file 41256_2021_205_MOESM1_ESM.docx]

| Search terms | Database searched |
| --- | --- |
| TI, AB (displace* OR refugee*) AND TI, AB (zoonos* OR "zoonotic disease*" OR "infectious disease*" OR "communicable disease*") | PubMed |
| TI, AB (displace* OR refugee*) AND TI, AB (zoonos* OR "zoonotic disease*") |  |
| ("humanitarian emergenc*" OR "humanitarian cris*") AND (zoonos* OR "zoonotic disease*" OR "infectious disease*" OR "communicable disease*") |  |
| TI, AB ("humanitarian emergenc*" OR "humanitarian cris*") AND TI, AB (zoonos* OR "zoonotic disease*" OR "infectious disease*" OR "communicable disease*") |  |
| TI, AB (displace* OR refugee*) AND TI, AB ("respiratory disease*" OR "vector borne disease*" OR leishmania* OR tuberculos* OR brucellosis OR rabies) |  |
| ("humanitarian emergenc*" OR "humanitarian cris*") AND ("respiratory disease*" OR "vector borne disease*" OR leishmania* OR tuberculos* OR brucellosis OR rabies) |  |
| (displace* OR refugee*) AND (zoonos* OR "zoonotic disease*) |  |
| ("humanitarian emergenc*" OR "humanitarian cris*") AND (zoonos* OR "zoonotic disease*" OR "infectious disease*" OR "communicable disease*") AND (framework* OR model*) |  |
| TI, AB (zoonos* OR "zoonotic disease*) AND TI, AB dynamic* |  |
| (displace* OR refugee*) AND (zoonos* OR "zoonotic disease*" OR "infectious disease*" OR "communicable disease*") AND (framework* OR model*) |  |
| (displace* OR refugee*) AND (animal* OR livestock) AND (zoonos* OR "zoonotic disease*" OR "infectious disease*" OR "communicable disease*") |  |
| ("humanitarian emergenc*" OR "humanitarian cris*") AND (animal* OR livestock) |  |
| (displace* OR refugee*) AND (animal* OR livestock) AND (disaster* OR conflict*) |  |
| (zoonos* OR zoonotic disease*) AND (displace* OR refugee*) AND disaster |  |
| (displace* OR refugee*) AND (zoonos* OR "zoonotic disease*" OR "infectious disease*" OR "communicable disease*") TOPIC | Web of Science |
| (displace* OR refugee*) AND (zoonos* OR "zoonotic disease*" OR "infectious disease*" OR "communicable disease*") TITLE |  |
| ("humanitarian emergenc*" OR "humanitarian cris*") AND (zoonos* OR "zoonotic disease*" OR "infectious disease*" OR "communicable disease*") TOPIC |  |
| (displace* OR refugee*) AND ("respiratory disease*" OR "vector borne disease*" OR leishmania* OR tuberculos* OR brucellosis OR rabies) TITLE |  |
| ("humanitarian emergenc*" OR "humanitarian cris*") AND ("respiratory disease*" OR "vector borne disease*" OR leishmania* OR tuberculos* OR brucellosis OR rabies) TITLE |  |
| ("humanitarian emergenc*" OR "humanitarian cris*") AND ("respiratory disease*" OR "vector borne disease*" OR leishmania* OR tuberculos* OR brucellosis OR rabies) TOPIC |  |
| (displace* OR refugee*) AND (zoonos* OR "zoonotic disease*”) TOPIC |  |
| ("humanitarian emergenc*" OR "humanitarian cris*") AND (zoonos* OR "zoonotic disease*" OR "infectious disease*" OR "communicable disease*") AND (framework* OR model*) TOPIC |  |
| (zoonos* OR "zoonotic disease*") AND dynamic* TITLE |  |
| (displace* OR refugee*) AND (zoonos* OR "zoonotic disease*" OR "infectious disease*" OR "communicable disease*") AND (framework* OR model*) TOPIC |  |
| (displace* OR refugee*) AND (animal* OR livestock) AND (zoonos* OR "zoonotic disease*" OR "infectious disease*" OR "communicable disease*") TOPIC |  |
| ("humanitarian emergenc*" OR "humanitarian cris*") AND (animal* OR livestock) TOPIC |  |
| ((displace* OR refugee*) AND (animal* OR livestock) AND (disaster* OR conflict*)) TOPIC |  |
| (displace* OR refugee*) AND (zoonos* OR "zoonotic disease*" OR "infectious disease*" OR "communicable disease*") | Plos |
| ("humanitarian emergenc*" OR "humanitarian cris*") AND (zoonos* OR "zoonotic disease*" OR "infectious disease*" OR "communicable disease*") |  |
| (displace* OR refugee*) AND ("respiratory disease*" OR "vector borne disease*" OR leishmania* OR tuberculos* OR brucellosis OR rabies) |  |
| ("humanitarian emergenc*" OR "humanitarian cris*") AND ("respiratory disease*" OR "vector borne disease*" OR leishmania* OR tuberculos* OR brucellosis OR rabies) |  |
| (displace* OR refugee*) AND (zoonos* OR "zoonotic disease*) |  |
| zoonoses in displacement |  |
| zoonoses AND displacement |  |
| ("humanitarian emergenc*" OR "humanitarian cris*") AND (zoonos* OR "zoonotic disease*" OR "infectious disease*" OR "communicable disease*") AND (framework* OR model*) |  |
| (displace* OR refugee*) AND (zoonos* OR "zoonotic disease*" OR "infectious disease*" OR "communicable disease*") AND (framework* OR model*) |  |
| zoono* AND displac* AND dynamic |  |
| (displace* OR refugee*) AND (zoonos* OR "zoonotic disease*") | ProQuest |
| ("humanitarian emergenc*" OR "humanitarian cris*") AND (zoonos* OR "zoonotic disease*") |  |
| (displace* OR refugee*) AND (animal* OR livestock) AND (zoonos* OR ("zoonotic disease" OR "zoonotic diseases")) |  |
| (("humanitarian emergencies" OR "humanitarian emergency") OR ("humanitarian crises" OR "humanitarian crisis")) AND (animal* OR livestock) AND (zoonos* OR ("zoonotic disease" OR "zoonotic diseases") OR ("infectious disease" OR "infectious diseases") OR ("communicable disease" OR "communicable diseases")) |  |
| (displaced OR displacement) AND (zoonoses OR zoonosis OR "zoonotic disease") | Science Direct |
| (displaced OR displacement) AND (animal OR livestock) AND (zoonoses OR zoonosis OR "zoonotic disease") |  |
| ("humanitarian emergency" OR "humanitarian crisis") AND (animal OR livestock) AND (zoonoses OR "zoonotic disease" OR "infectious disease" OR "communicable disease") |  |
| (displace OR refugee) AND (zoonoses OR "zoonotic disease") AND (framework OR model) |  |
| ((ab:(animal OR livestock) AND (displace* OR refugee*)) AND (disaster*)) | JSTOR |
| ((ab:(animal OR livestock) AND (displace* OR refugee*)) AND (zoonos* OR zoonotic disease*) AND (disaster*)) |  |
| (displace* OR refugee*) AND (zoonos* OR "zoonotic disease*") |  |
| zoonoses | Social Science Research Network |
| Displacement [search within: health] |  |
| BOOKS/ JOURNALS | Literature |
| (zoonos* OR zoonotic disease*) AND (displace* OR refugee*) AND disaster | Google Scholar |
| TI, AB (animal* OR livestock) AND (displace* OR refugee*) AND disaster |  |
